# CD40/anti-CD40 antibody complexes which illustrate agonist and antagonist structural switches

**DOI:** 10.1186/s12860-019-0213-4

**Published:** 2019-08-05

**Authors:** Maria A. Argiriadi, Lorenzo Benatuil, Ievgeniia Dubrovska, David A. Egan, Lei Gao, Amy Greischar, Jennifer Hardman, John Harlan, Ramesh B. Iyer, Russell A. Judge, Marc Lake, Denise C. Perron, Ramkrishna Sadhukhan, Bernhard Sielaff, Silvino Sousa, Rui Wang, Bradford L. McRae

**Affiliations:** 1 AbbVie Bioresearch Center, 381 Plantation Street, Worcester, MA 01605 USA; 2 AbbVie Bioresearch Center, 100 Research Drive, Worcester, MA 01605 USA; 3 AbbVie Inc., 1 North Waukegan Road, North Chicago, IL 60064 USA

**Keywords:** CD40, Crystal structure, Agonist, Antagonist, Antibody

## Abstract

**Background:**

CD40 is a 48 kDa type I transmembrane protein that is constitutively expressed on hematopoietic cells such as dendritic cells, macrophages, and B cells. Engagement of CD40 by CD40L expressed on T cells results in the production of proinflammatory cytokines, induces T helper cell function, and promotes macrophage activation. The involvement of CD40 in chronic immune activation has resulted in CD40 being proposed as a therapeutic target for a range of chronic inflammatory diseases. CD40 antagonists are currently being explored for the treatment of autoimmune diseases and several anti-CD40 agonist mAbs have entered clinical development for oncological indications.

**Results:**

To better understand the mode of action of anti-CD40 mAbs, we have determined the x-ray crystal structures of the ABBV-323 (anti-CD40 antagonist, ravagalimab) Fab alone, ABBV-323 Fab complexed to human CD40 and FAB516 (anti-CD40 agonist) complexed to human CD40. These three crystals structures 1) identify the conformational CD40 epitope for ABBV-323 recognition 2) illustrate conformational changes which occur in the CDRs of ABBV-323 Fab upon CD40 binding and 3) develop a structural hypothesis for an agonist/antagonist switch in the LCDR1 of this proprietary class of CD40 antibodies.

**Conclusions:**

The structure of ABBV-323 Fab demonstrates a unique method for antagonism by stabilizing the proposed functional antiparallel dimer for CD40 receptor via novel contacts to LCDR1, namely residue position R32 which is further supported by a closely related agonist antibody FAB516 which shows only monomeric recognition and no contacts with LCDR1 due to a mutation to L32 on LCDR1. These data provide a structural basis for the full antagonist activity of ABBV-323.

**Electronic supplementary material:**

The online version of this article (10.1186/s12860-019-0213-4) contains supplementary material, which is available to authorized users.

## Background

CD40 is a 48 kDa type I transmembrane protein that is expressed on a wide range of hematopoietic (B cells, monocytes/macrophages, dendritic cells) and non-hematopoietic (activated epithelium, endothelium) cells. Its ligand, CD154 or CD40L, has a more restricted expression pattern and is found primarily on activated T cells, B cells, and platelets. Engagement of CD40 by CD40L results in the recruitment of TNF receptor associated factors (TRAFs) to the cytoplasmic domain of CD40 [1]. Phosphorylation of various TRAF proteins results in activation of both canonical and non-canonical NF-kB pathways. TRAF6-dependent PI3K activation is a critical survival signal while TRAF2/TRAF6 have redundant functions in NF-kB activation and upregulation of CD80 and ICAM-1 expression [2]. Much of our understanding of CD40/CD40L biology comes from the interaction between antigen presenting cells [CD40 expression on either dendritic cells (DC) or B cells] and CD40L-expressing T cells. Cell-cell interactions between antigen presenting cells and T cells provide bidirectional signaling that is critical for the activation, maturation, and effector function of both cell types.

CD40 expression on epithelium, leukocytes, and vascular endothelium is elevated in organ-specific autoimmune diseases such as Crohn’s disease, ulcerative colitis, and rheumatoid arthritis and in systemic autoimmunity such as systemic lupus erythematosus (SLE). In addition, CD40+ monocytes, dendritic cells, and B cells are enriched at sites of chronic inflammation. Disruption of this signaling pathway has the potential to reduce production of proinflammatory cytokines, reduce T helper cell function, and inhibit macrophage activation, making it a very attractive therapeutic target for patients with chronic inflammatory disease [3]. X-ray structural studies have reported the CD40L-CD40 complex where 2 CD40 receptor monomers were bound to the intersubunit grooves of the CD40L trimer [4]. This protein-protein association was in part enabled by the receptor’s long and flexible structural fold containing 3 tandem cysteine-rich domains (CRDs). Each CD40 monomer associated within the CD40L intersubunit groove through variety of polar and hydrophobic interactions. Charge complementarity specifically played a large role in this association. An antibody that would prevent these interactions would act as an antagonist.

Attempts to disrupt the CD40-CD40L pathway for the treatment of autoimmunity and transplant rejection have been limited due to safety issues linked to the functional properties of specific biologic therapies. Two mAb programs (BG9588, Biogen; IDEC-131, IDEC) targeting CD40L entered clinical development for Systemic Lupus Erythematosis (SLE) and Crohn’s disease [3]. Development of both BG9588 and IDEC-131 was halted after multiple cases of thrombosis were reported. Subsequent studies suggested that antibodies against CD40L expressed on platelets may be cross-linked by FcγR also on platelets resulting in platelet degranulation and aggregation [5, 6]. Antibodies directed against CD40 such as BMS-224819 have been shown to block CD40-CD40L binding but have partial agonist activity resulting in some signaling through CD40 and peripheral B cell depletion [7]. In addition to B cell depletion, antibodies with CD40 agonist activity produce increases in liver enzymes and cytokine release that present safety concerns in patients with autoimmune disease [8]. Due to the various functional properties of anti-CD40 mAbs, it was critical to understand the molecular interactions of anti-CD40 mAbs with CD40. Therefore, we determined the crystal structures of ABBV-323 (antagonist) Fab alone and ABBV-323 and FAB516 (agonist) Fabs complexed to the 3 extracellular domains of human CD40 along with in vitro functional assays to better understand the protein interactions that govern agonist and antagonist activity of CD40 mAbs. Here we describe the molecular interactions that produce the full antagonist activity of ABBV-323. ABBV-323 (ravagalimab) has completed phase 1 testing in healthy volunteers and is currently entering phase 2 studies for the treatment of ulcerative colitis.

## Results

To identify CD40 specific antibodies, hybridoma technology was applied by immunizing mice with human CD40 antigen and adjuvant. Hybridoma antibodies were screened based on the following criteria: 1) human CD40 binding and neutralization of CD40 (EC_50_ < 10 nM), 2) no evidence of agonist activity in reporter assays or on primary human cells (> 100 nM), 3) retention of potent antagonist activity after expressing VH and VL as human chimeric antibodies, and 4) cross-reactivity to cynomolgus monkey CD40.

One chimeric antibody from the primary screen was selected for humanization, and affinity and liability engineering resulting in the lead mAb. Several variants of the humanized lead were produced by introducing semi-rational mutations in HCDR2. An HCDR2 S56G mutation variant was chosen to move forward due to a 10-fold improvement in affinity. Leucine to alanine mutations in the Fc region (234/235) were introduced to minimize FcgR binding and Fc-mediated effector function. Mutations that enhanced FcRn binding (T250Q, M428 L) were introduced to increase antibody half-life. We used a CD40-dependent NF-kB reporter assay to screen for potent CD40 antagonist. This system used recombinant CD40L to stimulate HEK-293 transfected with human CD40. NF-kB activation drove SEAP production which was quantitated using spectrophotometry. This was a very sensitive assay of both antagonist activity (ability to inhibit CD40-mediated signaling) and agonist activity (ability of the anti-CD40 mAbs to stimulate cells through CD40). Our lead mAb, ABBV-323, is a human IgG1/κ (hCg1_z,non-a allotype) antibody with minimum FcγR binding and Fc-mediated effector function due to the introduction of Leucine to alanine mutations in the Fc region (234/235) [9]. It also contains mutations that enhance FcRn binding (T250Q, M428 L) to increase antibody half-life [10]. ABBV-323 was a potent CD40 antagonist as measured in this reporter assay (EC_50_ = 3.7 nM) and showed no evidence of agonist activity in the absence of CD40L (data not shown).

**Fig. 1:**
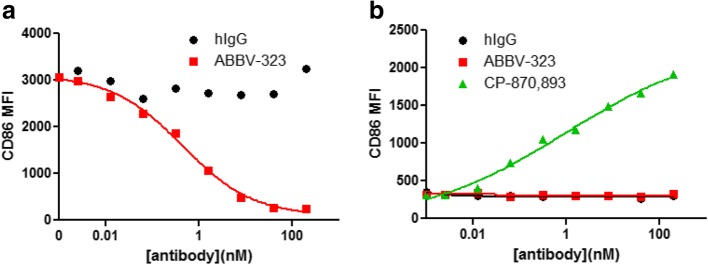
**a** and **b**: (**a**) ABBV-323 strongly inhibits CD40 signaling in B cells (inhibition of CD86 expression) without inducing agonist activity (stimulation of CD86 expression). To measure antagonist activity, CD40L+ jurkat cells were used to stimulate primary human B cells +/− ABBV-323. (**b**) To measure agonist activity, B cells were incubated with anti-CD40 antibodies

We developed a primary B cell assay to test the antagonist and agonist activity of anti-CD40 mAbs in a more physiologically relevant system. B cells express the highest levels of CD40 of any cell type in human peripheral blood and also express FcγR which allowed us to evaluate potential for residual FcγR binding in promoting CD40 agonist activity. To measure antagonist activity, Jurkat cells were used as a source of native CD40L and CD40-dependent B cell activation was measured by cell proliferation and induction of CD86 by flow cytometry. ABBV-323 inhibited CD86 upregulation on primary human B cells with an EC_50_ value of 0.6 nM (Fig 1a). In the agonist assay a known CD40 agonist, CP-870,893 [11], dose-dependently induced CD86 expression on B cells while ABBV-323 showed no evidence of agonist activity (Fig. 1b).

Receptor crosslinking is known to be critical to initiate downstream signaling for TNF receptor family members. Although Fc crosslinking was suggested to play an important role under certain conditions, it was shown to be not essential for agonizing CD40 receptor (Richman, et al. Cancer Immunol Res. 2014) [12–14]. On the contrary, we have demonstrated that the antibody binding epitope plays an important role in function and that the Fc crosslinking is not required for agonist activity.

**Fig. 2:**
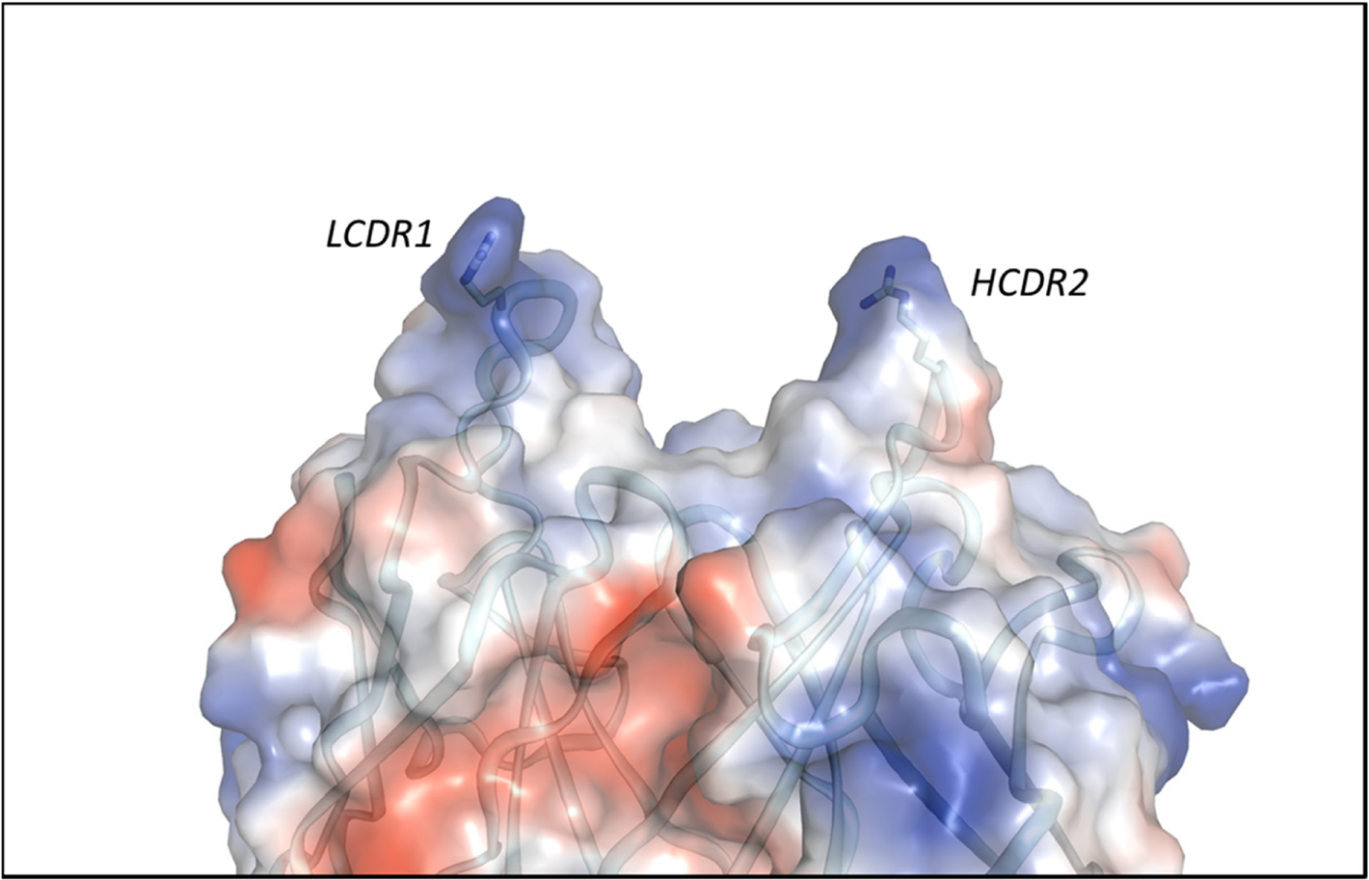
Electrostatic potential surface calculated in Pymol for Fab ABBV-323 crystal structure. A cleft is formed between HCDR2 and LCDR1. Red patches refer to negative charged regions and blue patches refer to positive charged regions

**Fig. 3:**
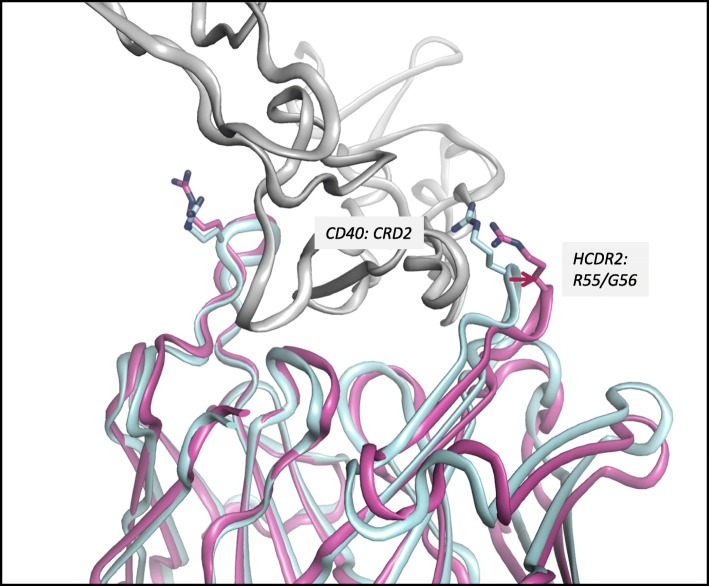
Complex structure of ABBV-323 Fab (magenta) and human CD40 (shown in grey). The Fab alone structure is superimposed (in cyan) to show how HCDR2 opens to accommodate CD40

A crystal structure of ABBV-323 Fab was solved to 1.74 Å resolution and the ABBV-323 Fab/CD40 complex structure was solved to 2.84 Å resolution. Critical observations include the identification of the 3-dimensional conformational epitope of ABBV-323 and the key structural rearrangement needed for its Fab to accommodate the CD40 antigen. The structure of ABBV-323 Fab alone illustrates two loops (HCDR2 and LCDR1) which create a cleft of approximately 25 Å with a diversity of charge (Fig. 2). The structure of ABBV-323 Fab bound to CD40 exhibits two complexes in the asymmetric unit. Within the asymmetric unit, each complex contains one CD40 monomer associated with the Fab on CRD2 (Fig. 3). When overlaying the two complexes within the repeating crystal unit, there is a slight shift in CRD3 due to the inherent flexibility of the cysteine rich domains, however the epitope contacts to CRD2 are constant. When comparing the complex structure with the Fab alone structure, there is a backbone shift in HCDR2 that allows opening of the cleft to accommodate CD40 binding (Fig. 3). As described earlier, mutation data supports this structural observation: a HCDR2 S56G residue change results in 20-fold improvement in binding affinity due to higher flexibility introduced by a glycine at this position. This flexibility most likely aids Fab cleft opening and facilitates a hydrogen bond interaction of (H)R55 with CD40.

**Fig. 4:**
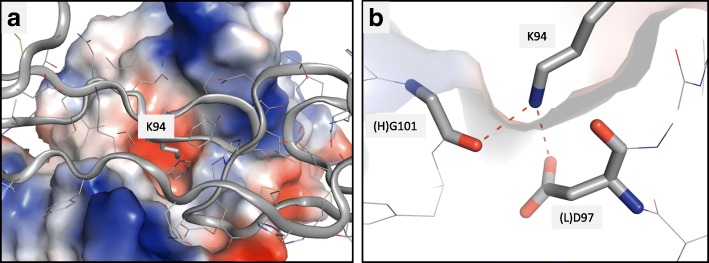
**a** and **b**: (**a**) K94 inserts into a negatively charged channel to make interactions with acidic pocket. (**b**) Interactions between K94 and (H)G101/(L)D97 are illustrated

The conformational epitope of ABBV-323 shows several critical interactions. Specifically, CD40 residue K94 inserts into the central cleft of ABBV-323 and specifically binds to a negatively charged region outlined by residues (L)D97 and the backbone carbonyl of (H)G101 (Fig. 4a and b). Additional epitope interactions are observed for both heavy and light chains: CD40 E64 displays hydrogen bonds to (H)S53/(H)Y50 and CD40 T99 displays a hydrogen bond to (L)N31. When overlaying the complex structure with a previously reported structure of CD40/CD40L (PDB code: 3QD6 [4]), the CD40 antigens adopt similar conformations in the region of CRD1 and CRD2. From the superposition, the Fab HCDR2 would minimally clash with several residues on an external loop of CD40L (amino acid residues S128-K143). Therefore, it is conceivable that this loop could move to accommodate simultaneous binding of CD40L, CD40 monomer and Fab in an agonist-like ternary complex.

**Fig. 5:**
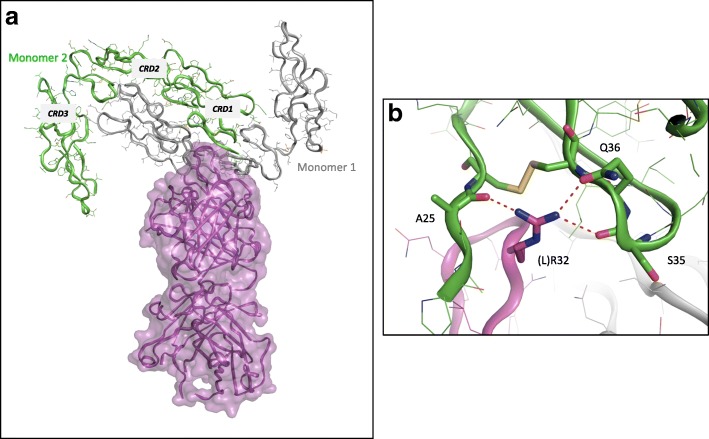
**a** and **b**: (**a**) Two crystallographic CD40 monomers associate to form a tight antiparallel dimer (grey, green) which bind to ABBV-323 Fab (shown in magenta). (**b**) LCDR1 R32 inserts in the second crystallographic monomer shown in green. R32 makes interactions with backbone carbonyls to A25/S35 and sidechain Q36

When generating symmetry crystallographic equivalents of the CD40/ABBV-323 Fab complex, CD40 is observed as a tight antiparallel homodimer (Fig. 5a). The interface of the homodimer spans approximately 40 Å long with extensive hydrogen bond complementarity and VDW contacts, and a buried surface area of 3292.5 Å^2^. Additionally, a PISA analysis predicts this homodimeric interface to be stable in solution with a calculated ΔG_diss_ = 24.3 kcal/mol. Not only is ABBV-323 bound to the primary monomer (“monomer 1”), a second monomer (“monomer 2”) of the crystallographic dimer associates with ABBV-323 through the N-terminal end of CD40’s CRD1 which includes the amino acid residues T24-C37. Specifically, a position off of the Fab’s LCDR1, (L)R32 bridges two strands of CD40 CRD1 contacting backbone carbonyls from CD40 (monomer 2) A25/S35 and a potential sidechain interaction with Q36 (Fig. 5b). This observation led to a structural hypothesis that ABBV-323 recognizes a biologically relevant CD40 “inactive” antiparallel homodimer.

The concept behind a CD40 functional dimer is also supported by a previous report where CD40 1) demonstrated homodimerization in soluble and cell surface-expressed environments, and 2) CD40 dimerization was dependent on the extracellular domain [15]. Additionally, a homology model was created of the homodimer based on the ligand-free TNFR1 structure and mutations in the CRD1 domain of CD40 were made based on the dimeric model. When mutating K29 (a key dimerization residue in the model) to a non-polar or acidic residue (K29A and K29E), self-assembly decreased [15]. In our reported crystal structure of ABBV-323 Fab complexed to CD40, K29 participates in the observed antiparallel crystallographic dimer. Specifically, K29 is proximal to the dimeric interface and makes a potential interaction with a nearby residue from the second CD40 monomer E28. It is likely that these two residues engage in a stabilizing hydrogen bond interaction for the CD40 homodimer. Therefore, mutating this residue position to a non-polar or acidic residue could negatively affect self-assembly which requires charge complementarity for dimerization.

**Fig. 6:**
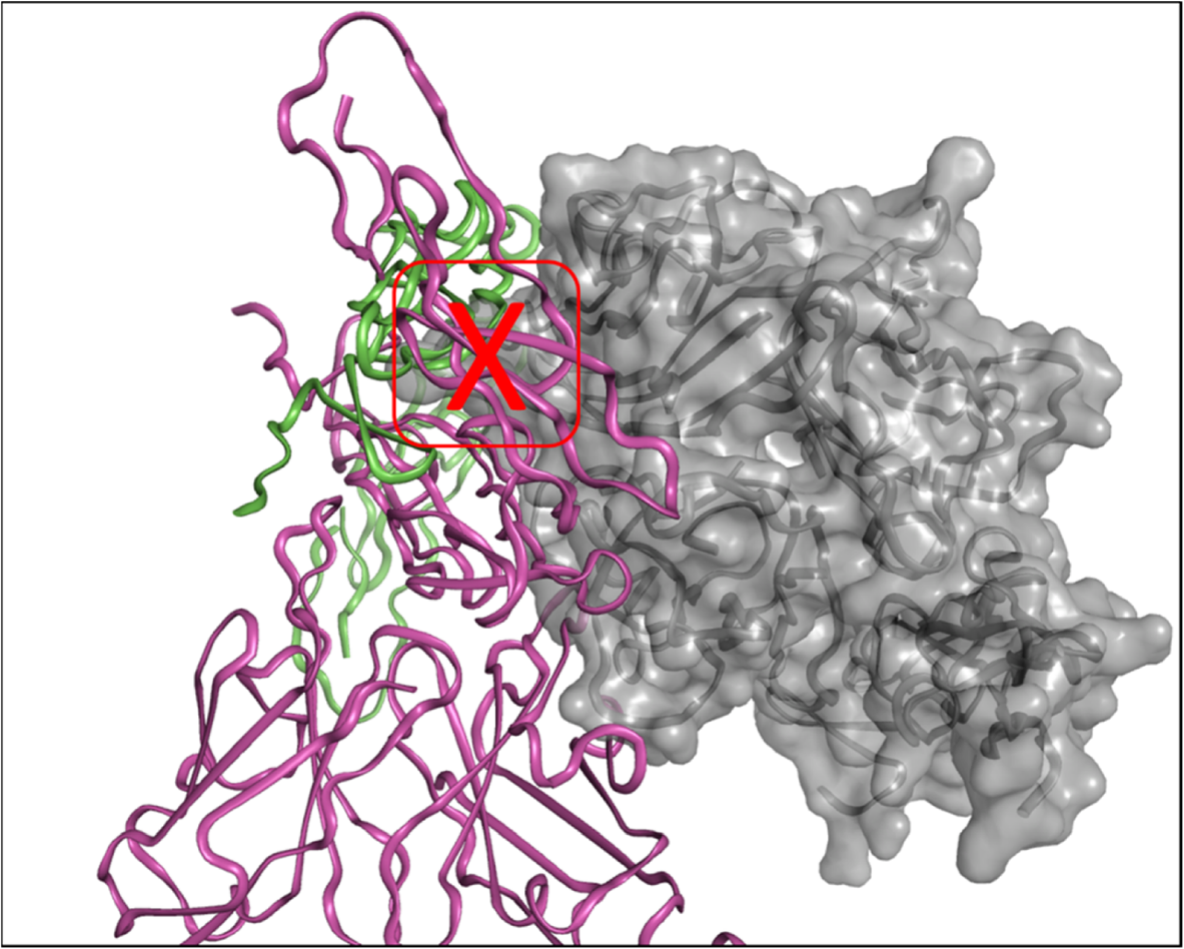
Overlay of ABBV-323/CD40 complex (magenta = Fab/CD40 monomer 1, green = CD40 crystallographic monomer 2) with crystal structure of CD40L/CD40 complex (PDB code: 3QD6 in grey surface) [4]. Red outlined box shows the region of significant steric clash between CD40 dimer and CD40L

Structural insights from previous reports suggest that unliganded TNF family receptors dimerize in antiparallel orientations between the first two disulfide-rich motifs of the receptor thereby forming a non-signaling structural state [16, 17]. This is potentially a similar scenario for CD40 receptor which is a member of the TNFα superfamily. Antiparallel CD40 homodimers would prevent downstream cytoplasmic signaling proteins to bind to CD40 receptor because 1) CD40 antiparallel dimers would occlude the CD40L binding site which would then 2) prevent CD40 from clustering to recruit TRAF adaptor proteins that activate different signaling pathways [13]. The co-crystal structure of ABBV-323 Fab/CD40 supports this idea. When comparing the CD40 antiparallel dimer with a previously reported TNFR1 antiparallel dimer, the antiparallel recognition is different: CD40 dimerizes with a larger buried surface area via the CRD1–2 domains while TNFR1 dimerizes primarily through the CRD2 domain with a smaller buried dimeric surface area (1475 Å2, Additional file 1: Figure S1) [16]. When overlaying the ABBV-323 Fab complex structure (including the crystallographic antiparallel homodimer) with the historical structure of CD40/CD40L (PDB code 3QD6 [4]), the superposition demonstrates that CD40L would be unable to bind the antiparallel homodimer of CD40 due to steric clashes spanning approximately 16 Å, specifically in the CD40L loop region L195-I205 (Fig. 6). We therefore conclude that ABBV-323 functions as a CD40 antagonist antibody due to its ability to capture the non-signaling dimeric state of CD40 receptor which sterically masks CD40L recognition. A better understanding of the molecular basis for agonist and antagonist activity of anti-CD40 antibodies is critical for the rational design of therapeutics with the desired efficacy and tolerability profile.

**Fig. 7:**
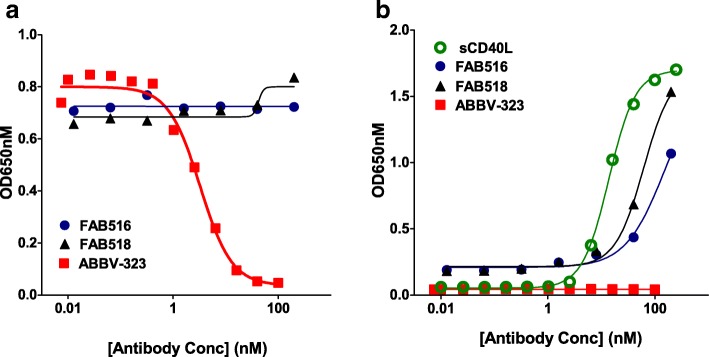
**a** and **b**: (**a**) ABBV-323 was incubated with HEK-293 CD40L cells in the presence of CD40L expressing D1.1 cells. The ability of ABBV-323 to inhibit the interaction between CD40 and its receptor CD40L is monitored by the production of SEAP compared to an IgG control. (**b**) Agonist activity is monitored as above with the following exception: CD40L-expressing D1.1 cells were replaced with assay media. CD40L was titrated alongside ABBV-323 as a positive control (Concentration range = 250 ng/ml – 0.026 ng/ml)

To further support this idea of a non-signaling dimeric state, we examined several variants of ABBV-323 with small changes to sequence in LCDR1 including changing position (L)R32 to proline (FAB516) or leucine (FAB518). These changes resulted in conversion of the antibody from antagonist to agonist activity as demonstrated in a human CD40 reporter assay (Fig. 7a, b; Table 1). This supports the claim that the LCDR1 loop in ABBV-323 (specifically amino acid position R32) may be critical in binding the second monomer of CD40 through specific polar interactions and thereby stabilizing the inactive antiparallel homodimer. When changing this residue to a non-polar leucine or proline, this functional dimer would not form due to lack of polar contacts with LCDR1. We predicted that x-ray complexes of agonist variants such as FAB516 or FAB518 (which respectively have a proline ((L)P32) and leucine ((L)L32) in place of an arginine on LCDR1) would exhibit different crystallization packing and oligomerization in contrast to ABBV-323. To confirm this hypothesis, the crystal structure of agonist FAB516 Fab complexed to CD40 was solved to 3.1 Å resolution. The structure shows very clear electron density for the CD40/Fab interface and crystallographic packing is unambiguous. Comparison of the two antibody complexes (CD40/ABBV-323 and CD40/FAB516) was then needed to understand oligomerization of the receptor.

**Table 1:** Measuring agonist and antagonist activities of several CD40 antibody variants

Antibody variants	VL LCDR1 Sequence (KSSQSLLN**S**GNQKNYLT)	Blocking of CD40L	Agonist: huCD40 reporter assay IC50 nM	Antagonist: Jurkat/ Reporter assay IC50 nM
ABBV-323	KSSQSLLN**R**GNQKNYLT	Yes	No	3.1
FAB518	KSSQSLLN**P**GNQKNYLT	Yes	62	No
FAB516	KSSQSLLN**L**GNQKNYLT	Yes	157	No

**Table 2:** Crystallographic statistics

Structure	ABBV-323 Fab alone	CD40 complexed to ABBV-323	CD40 complexed to FAB516
PDB code	6PE7	6PE8	6PE9
Data Collection			
Resolution (Å)	132.6–1.74	126.9–2.84	167.5–3.13
Space Group	C222_1_	P2_1_2_1_2	C2
Unit Cell Lengths (a, b, c; Å) Angles (°)	63.7 130.4 132.6	173.3 76.0 126.1	254.8 224.0 111.4 β = 98.0
Unique reflections	56956	40335	108254
Overall Statistics (Highest Shell)			
R_merge_ (%)	0.041 (0.94)	0.11 (0.974)	0.108 (0.632)
I/σ_I_	25.5 (2.2)	15.8 (2.3)	11.2 (2.1)
Data completeness (%)	100 (100)	100 (100)	99.4 (98.6)
Mean multiplicity	6.6 (6.6)	6.6 (6.8)	3.4 (3.2)
CC(1/2)	1.00 (0.79)	1.00 (0.72)	0.99 (0.76)
Refinement			
Resolution (Å)	24.4–1.74	38.0–2.84	35.3–3.13
Reflections used in refinement	56860	40063	107879
R_cryst_ (%)	19.6	20.5	21.6
R_free_ (%)	23.0	26.5	25.5
R.m.s. deviations, bond lengths (Å), bond angles (°)	0.005, 0.865	0.010, 1.13	0.010, 1.13
Ramachandran Favored regions (%)	97.7	94.9	93.6
Outliers (%)	0.23	0.17	0.21

## Discussion

**Fig.8:**
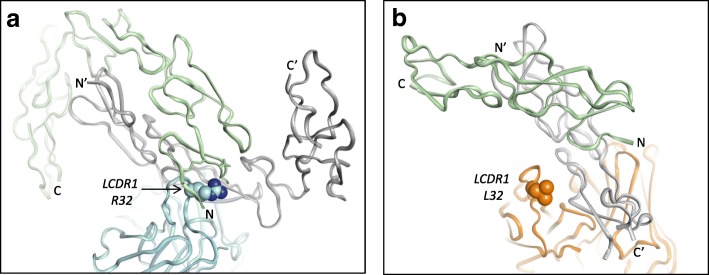
**a** and **b** (**a**) ABBV-323 Fab (blue) bound to crystallographic CD40 dimer (second monomer shown in green ribbon) which shows interaction with LCDR1 R32. (**b**) FAB516 Fab (orange) bound to crystallographic CD40 dimer (second monomer shown in green ribbon) which shows no interaction with LCDR1 L32. N and C termini are also labeled to demonstrate the antiparallel dimers for both ABBV-323 and FAB516

When overlaying the backbones of FAB516 and ABBV-323 CD40 complex structures, the CD40 monomer recognition for both Fabs is identical. However as predicted, the crystallographic dimer formation is different. When generating the symmetry equivalents of the FAB516 complex structure, an antiparallel CD40 dimer is again observed however the second CD40 monomer is in a different orientation when compared to the CD40 homodimer in the ABBV-323 complex structure (Fig. 8a and b). A PISA analysis for the FAB516-CD40 complex calculates the crystallographic CD40 homodimer interface to have a smaller buried surface area (1397.6 Å2) and a smaller predicted ΔG_diss_ (2.5 kcal/mol) when compared to the values calculated for the ABBV-323 complex suggesting that the FAB516 CD40 dimer interface is less physiologically relevant. Most importantly, the FAB516 Fab binds predominantly to one monomer of CD40. Specifically, LCDR1 (L)L32 does not engage in interactions with a second CD40 monomer (Fig. 8b) for 4 out of the 5 Fab-antigen complexes in the asymmetric unit. Due to these observations, we conclude that FAB516 recognizes CD40 monomer and not the functional homodimer as illustrated with the ABBV-323 complex.

**Fig. 9:**
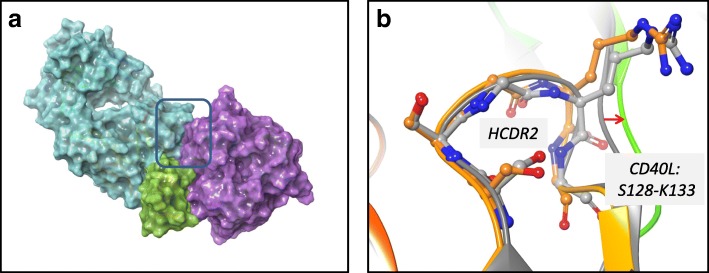
**a** and **b**: (**a**) Model of potential ternary complex of agonist FAB516 Fab (cyan), human CD40 (green), human CD40L (purple). Box outlines region where minor structural rearrangements may occur to accommodate agonist complex. (**b**) After ternary complex minimization in MAESTRO, HCDR2 (S52-G56) and CD40L (S128-K132) residues shift to accommodate the interface between CD40L, FAB516 and CD40

This difference in CD40 recognition supports the agonist activity seen for FAB516. When observing a superposition of the backbone structure of the FAB516 complex structure with the CD40L/CD40 complex structure (PDB code 3QD6 [4]), there are several clashes between the FAB516/CD40 monomeric complex and CD40L, specifically in the region of the heavy chain CDRs. This observation is in agreement with blocking results (Table 1). However, with some structural rearrangement, an agonist ternary complex (CD40/CD40L/FAB516, Fig. 9a and b) could also be hypothesized suggesting that FAB516 could act potentially as an agonist stabilizer. Specifically, when minimizing the proposed ternary complex, HCDR2 (residues S52-G56) and CD40L (residues S128-K132) conformationally adjust to create the complex. This concept is in agreement with reporter assay results in Table 1 (Fig. 7a and b).

We conclude that a non-polar residue at position LCDR1(32) as seen in FAB518 (proline) and FAB516 (leucine), allows agonist antibody recognition of monomeric CD40 and a potential ternary complex with CD40L. In contrast, when this position switches to a basic polar residue (specifically in the case of antagonist ABBV-323 (L)R32), the antibody recognizes an antiparallel dimeric CD40 which prevents CD40L binding.

## Conclusion

In this study, the structures of ABBV-323 Fab alone and ABBV-323/FAB516 Fabs in complex to CD40 were solved. The structure of ABBV-323 demonstrates a unique method for antagonism by stabilizing the proposed functional antiparallel dimer for CD40 receptor via novel contacts to LCDR1, namely R32. This was further supported by a closely related agonist antibody FAB516 which showed only monomeric recognition and no contacts with LCDR1 due to a mutation to L32 on LCDR1. Therefore, a structural agonist/antagonist switch in this class of antibodies was identified. Antibodies for antiparallel dimers have also been recently reported for the TNFR2 receptor in the inhibition of proliferation of ovarian cancer cells [18]. In this report, a model was created demonstrating how the TNFR2 antiparallel dimer locked into its quiescent state via the antagonist antibody to prevent TNF binding and signaling. It was also hypothesized that these resting state TNFR2 dimers could also arrange into a higher order hexagonal lattice on the cell surface. Although we did not observe a hexagonal arrangement in the crystal packing of the ABBV-323/CD40 complex structure, it would be interesting to observe the nature of the CD40 resting state antiparallel dimers using orthogonal techniques such as SAXS or Cryo-EM with the potential of observing higher order CD40 oligomerization. In conclusion, our structural findings which demonstrate the ABBV-323 antibody’s mechanism of action suggest a growing strategy for designing and identifying antagonists to not only CD40 receptor but other members of the TNF superfamily.

## Methods

### Bioactivity measurement of CD40 antibodies – primary B cell assay

Primary human B cells were purchased from BioSpecialty and were used to study CD40 signaling. Cells were cultured in RPMI with 10% heat-inactivated serum, 2 mM L-glutamine, 1 mM sodium pyruvate and 100μg/ml Pen Strep. To measure agonist activity, the cells were plated at 0.1 million per well of a 96-well plate and dilutions of CD40 antibodies were incubated with the cells for 2 days. Cells were then harvested and CD86 upregulation was measured by FACS. To assess antagonist activity, B cells were incubated with CD40L-expressing Jurkat cells (ATCC CRL-10915) at 2:1 ratio per well of 96-well plate in the presence of CD40 antibodies. Cells were harvested 2 days later and stained for CD19, CD20 (B cell marker) and CD86. Briefly, cells were washed by PBS and incubated with antibodies in PBS with 2% serum for 20 min on ice. The cells were then washed by PBS and analyzed by a BD LSRFortessa™ cell analyzer. Anti-CD19 and anti-CD20 were purchased from BD bioscience and anti-CD86 was from eBiosciences. The ability of the anti-CD40 antibodies to inhibit the interaction between CD40 and its receptor CD40L was compared to an IgG control.

### HEK-293 blue CD40L – antagonist reporter assay

HEK-293 Blue CD40L Cells were seeded into 96-well flat bottom TC plates at a density of 5 × 10^4^ cells/100 μl/well. The plates were incubated for 1 h at 37 °C, 5% CO2. Stock solutions of CD40 antibodies were prepared in assay medium at a 4x concentration (400 nM), serially diluted 1:2.5 and added to HEK-293 cells (50 μl /well). CD40L-expressing D1.1 cells were diluted to a concentration of 1 × 10^6^/ml in assay media and added to all wells (50 μl /well) bringing the final Ratio of D1.1 to HEK-293 to 1:1. Plates were incubated for 18 h at 37 °C, 5% CO2. Following incubation, 50 μL of supernatant media was removed and transferred to a new plate for QUANTI-Blue luminescence detection. IC50 values were obtained using logarithm of antibody concentration vs. luminescence nonlinear regression (4-parameter dose-response curve model:

$$ {\displaystyle \begin{array}{l}\mathrm{Y}=\mathrm{Bottom}+\left(\mathrm{Top}\hbox{-} \mathrm{Bottom}\right)/\left(1+{10}^{\wedge}\left({\left(\mathrm{LogIC}50\hbox{-} \mathrm{X}\right)}^{\ast}\left.\mathrm{HillSlope}\right)\right)\right)\kern0.5em \mathrm{in}\kern0.5em \mathrm{GraphPad}\kern0.5em \mathrm{Prism}\kern0.5em 5.\mathrm{Three}\ \\ {}\mathrm{in}\mathrm{dependent}\kern0.5em \mathrm{experiments}\kern0.5em \mathrm{were}\kern0.5em \mathrm{performed}.\end{array}} $$

### HEK-293 blue CD40L – agonist reporter assay

Assay performed as above with the following exception: CD40L-expressing D1.1 cells were replaced with 50 μl assay media. A 4x stock solution of recombinant human MegaCD40L (positive control) was prepared at 1 μg/ml in assay media and serially diluted 1:2.5. Dilutions were added alongside CD40 antibodies. Concentration range = 250 ng/ml – 0.026 ng/ml.

### Preparation and purification of CD40 antigen

A DNA sequence encoding the human CD40 extracellular domain (UNIPROT P25942; amino acids 1–193) was synthesized and cloned into our proprietary pHybE (US Patent 8187836 B2) vector followed by an in-frame C-terminal Tev protease cleavage site and hexahistidine tag (sequence ENLYFQGHHHHHH). The pHybE expression vector utilizes an EF-1α promoter and an OriP origin of replication derived from Epstein-Barr virus (EBV). This plasmid was transfected into HEK-293-6E cells (NRC Canada) grown in Freestyle 293 medium (Thermo Fischer) at 1 × 10^6^ cells/ml using the transfection reagent Polyethylenimine (PEI, Polysciences Inc) at a PEI:DNA ratio of 4:1. The transfected cell culture was fed with tryptone-N1 (Organotechnie) (to 0.5%) at 24 h post-transfection. On day 7 post-transfection, the transfected cell culture was cleared by centrifugation followed by filtration through 0.2 μm PES filter (Corning). Cleared medium was buffer exchanged to PBS, pH 7.4 using a Kvick TFF system equipped with 10 kDa membranes (GE Healthcare) and loaded on a 5 ml HisTrap FF column (GE Healthcare) equilibrated with PBS, pH 7.4. The column was washed with 25 mM imidazole in PBS, pH 7.4 and bound protein was eluted with 250 mM imidazole in PBS, pH 7.4. Eluted protein was concentrated using Amicon Ultra-15 centrifugal filter devices (Millipore) with 10 kDa molecular weight cut-off, and further purified by SEC on a 26/60 Superdex 200 column (GE Healthcare) equilibrated and run with PBS, pH 7.4. Fractions containing CD40 were pooled, concentration measured by absorbance at 280 nm, and samples analyzed by SEC, SDS-PAGE, and mass spectrometry. [CD40(h)(21–193)]-Tev-His6 was stored in aliquots at − 80 °C.

### Preparation and purification of CD40 ABBV-323 Fab fragment

Fab fragment of CD40 ABBV-323 was prepared by papain cleavage of the parent mAb ABBV-323 as detailed below. Papain (Worthington Biochemical, Lakewood, NJ) was activated with 50 mM cysteine in PBS, pH 7.4 buffer. The parent mAb in PBS, pH 7.4 buffer was mixed with papain at 1:100 weight ratio of papain to mAb and incubated for 1 h at 37 °C. The reaction was quenched with 5 mM iodoacetamide. The mixture was purified on 10 ml Mab SelectSure resin (GE Healthcare) where the Fab fragment was collected as flow through. The flow through was concentrated using an Ultrafree-15 Biomax 10 kDa molecular weight cut-off (MWCO) centrifugal device (Millipore). The concentrated mixture was purified on 2.6 cm × 60 cm Sephacryl 200 HiPrep column (GE Healthcare) pre-equilibrated in 50 mM HEPES, 50 mM NaCl, pH 7.5 buffer. Fractions containing Fab fragment (monitored by UV absorbance at 280 nm) were pooled and frozen at − 80 °C. Sample purity was assessed by analytical SEC, SDS-PAGE and mass spectrometry. Table 3 lists protein quality characterizations for the reagents used in this study.

**Table 3:** Protein quality characterization

Protein name	Isotype		Monomer % by SEC	Endotoxin level^a^	Mass Spec. Identity^b^
	Heavy chain	Light chain			
FAB518	hCg1_z,non-a	Kappa	100	< 0.1 EU/mg	Consistent
FAB516	hCg1_z,non-a	Kappa	100	< 0.1 EU/mg	Consistent
CP 870,893	hCg1_z,non-a	Kappa	99	< 0.1 EU/mg	Consistent
ABBV-323	hCg1_z,non-a	Kappa	100	< 0.4 EU/mg	Consistent

^a^Endotoxin levels assessed by PTS EndoSafe LAL

^b^Expected (theoretical) molecular weight (MW) is consistent with measured MW by Mass Spec

### CD40/CD40 ABBV-323 Fab complex preparation

Recombinant human CD40 was expressed and purified as described above. Recombinant human CD40 and CD40 ABBV-323 Fab protein were mixed at a 1.05:1 M ratio (1.8 mg/ml final concentration) and incubated for 4 h at 4 °C. The complex sample was loaded onto a 2.6 cm × 60 cm Sephacryl 200 HiPrep column (GE Healthcare) pre-equilibrated in 50 mM HEPES, 50 mM NaCl, pH 7.5 buffer at 1 ml/min. Fractions containing the complex (monitored by UV absorbance at 280 nm) were pooled and concentrated to 18 mg/ml using an Ultrafree-15 Biomax 10 kDa molecular weight cut-off (MWCO) centrifugal device (Millipore). Sample purity was assessed by analytical SEC and SDS-PAGE. Excess Fab-Complex protein was stored frozen at − 80 °C.

### ABBV-323 Fab crystallization

Fab alone was supplied at 22.5 mg/ml in 50 mM HEPES, 50 mM NaCl, pH 7.5. Crystals grew by vapor diffusion at 23 °C. The reservoir contained 25% (w/v) PMME 550, 0.1 M MES pH 6.5, 0.01 M zinc sulfate. The drop was made by adding equal volumes of protein and reservoir solution. Crystals grew as thick prisms and were cryo-protected using the reservoir solution with the addition of 10%(v/v) propylene glycol. Crystals were harvested, swished through cryo-solution and cryo-cooled directly in liquid nitrogen. Diffraction data to 1.74 Å were collected under gaseous nitrogen at 100 K at the 17ID beamline at the Advanced Photon Source at Argonne National Laboratories (Argonne IL).

### ABBV-323 Fab complexed to CD40 antigen crystallization

The Fab complex was supplied at 18 mg/ml in 50 mM HEPES, 50 mM NaCl, pH 7.5. The antigen construct used was [CD40 (h) (21–193)]-TEV-6His. Crystals grew by vapor diffusion at 23 °C. The reservoir contained 2 M ammonium sulfate, 0.1 M phosphate-citrate pH 4.2. The drop was made by adding equal volumes of protein and reservoir solution. Crystals grew as thin rods and were cryo-protected using 2.5 M lithium sulfate. Crystals were harvested, swished through cryo-solution and cryo-cooled directly in liquid nitrogen. Diffraction data to 2.84 Å were collected under gaseous nitrogen at 100 K at the 17ID beamline at the Advanced Photon Source at Argonne National Laboratories (Argonne IL).

### Preparation of FAB516 complex by direct co-expression

#### Fermentation

The expression plasmid (pHybE) for heavy chain of FAB516 was synthesized with secretion leader sequence, variable region, CH1 and hinge region (ending at H224, Eu numbering). The light chain plasmid (pHybE) was synthesized with secretion leader sequence, variable region and CL region (ending at C214, Eu numbering). The plasmid for antigen expression was constructed as described above. HEK-293-6E cells, which is a suspension adapted human embryonic kidney-293-based cell line stably expressing the Epstein–Barr virus nuclear antigen (EBNA1), were transfected with plasmid DNA encoding the heavy chain (HC) and light chain (LC) of FAB516 and plasmid for the CD40 antigen ([CD40(h)(1–193)]-TEV-6His). For a 3 L expression, a 5 L flask (Thompson Instrument Company) containing cells at 1.2 × 10^6^ cells/flask were grown in Freestyle 293 Expression medium at a temperature of 37 °C, with 8% CO2, and shaking at 80 rpm. For transfection, 1.5 mg of DNA in 1:2:3 ratio (HC:LC:Antigen) was mixed with 6 ml of 1 mg/ml pH 7.0 PEI solution in a volume of 150 ml Freestyle medium. After 10 min of incubation, the mixture was added to the cells. Tryptone N1 (Organotechnie) in Freestyle medium was added to the flask at 24 h post transfection for 0.5% final concentration. The conditioned medium was harvested 9–11 days post transfection by centrifugation at 16 K x G for 10 min, followed by clarification through a Pall AcroPak 500 0.8/0.45 μm filter capsule, and sodium azide was added from a 1 M stock to a concentration of 5 mM. The conditioned media was stored at 4 °C until purified.

#### CD40/CD40 FAB516 Fab complex preparation

The cell culture supernatant containing Fab/antigen complex was loaded onto a 22 ml Ni Excel (GE Life Sciences) column at a flow rate of 5 ml/min, and washed with 5 column volumes of binding buffer (50 mM Tris, 350 mM sodium chloride, pH 8.0). The non-specific proteins bound to the column were washed with 5 column volumes of binding buffer containing 20 mM imidazole. The Fab/antigen complex was eluted by gradient elution from 5 to 100% elution buffer (50 mM Tris, 300 mM sodium chloride 400 mM imidazole pH 8) in 10 column volumes. The Fab/antigen complex was collected in 7 ml fractions. Size-exclusion chromatography was performed as a polishing step using a HiLoad 26/60 Superdex 75 column (GE Life Sciences). The Superdex 75 column was run at 1 ml/min with 50 mM HEPES, 50 mM NaCl pH 7.5. Fractions containing Fab/antigen complex was characterized with SEC, SDS-PAGE gel and mass spec, and concentrated to 20.0 mg/ml for crystallography.

### FAB516 Fab complexed to CD40 antigen crystallization

The Fab complex crystals grew by vapor diffusion using sitting drop at 23 °C. The reservoir contained 1.6 M Ammonium Sulfate, 2% w/v PEG1000, 100 mM HEPES Sodium Salt pH 7.5 (JBS-II screen from Jena Bioscience, conditions A9). The drop was made by adding equal volumes of protein and reservoir solution (0.4 μL + 0.4 μL). Diffracting crystals took 11 months to appear and were cryo-protected using the reservoir solution with 20% Ethylene glycol. Crystals were harvested, swished through cryo-solution and cryo-cooled directly in liquid nitrogen. Diffraction data to 3.13 Å were collected under gaseous nitrogen at 100 K at the 17ID beamline at the Advanced Photon Source at Argonne National Laboratories (Argonne IL).

### Structure determinations of ABBV-323 Fab and CD40 antigen complexes

Diffraction data for both crystal structures were processed using the program AUTOPROC with isotropic scaling from Global Phasing Ltd. [19] The ABBV-323 Fab dataset was processed in the space group C222_1_ with the following unit cell dimensions: a = 64.65 b = 130.4 c = 132.6. A maximum likelihood molecular replacement solution was determined using the program PHASER [20] using a Fab search model reported previously (Protein Data Bank entry 3QOS [21]). Coordinates for 1 Fab molecule in the asymmetric unit were generated based on the molecular replacement solution. Preliminary refinement of the resulting solution was conducted using REFMAC [22, 23] and the program BUSTER [24]. Iterative protein model building was conducted using the program COOT [25] and examination of 2Fo-Fc and Fo-Fc electron-density maps. Water molecules were added using BUSTER refinement [24]. A final round of refinement was conducted using PHENIX [26]. Final refinement statistics reported R_free_/R_work_ values of 0.230/0.196.

The ABBV-323 Fab CD40 complex dataset was processed in the space group P2_1_2_1_2 with the following unit cell dimensions: a = 173.3 b = 76.0 c = 126.1. A maximum likelihood molecular replacement solution was determined using the program PHASER [20] using the previously solved ABBV-323 Fab reported above. Coordinates for 2 Fab molecules were found in the asymmetric unit based on the molecular replacement solution. Preliminary refinement of the resulting solution was conducted using REFMAC [22, 23] and the program BUSTER [24]. The model for CD40 was built manually using the program COOT [25] and examination of 2Fo-Fc and Fo-Fc electron-density maps. Water molecules were added using BUSTER refinement [24]. A final round of refinement was conducted using PHENIX [26]. Final refinement statistics reported R_free_/R_work_ values of 0.265/0.205.

The FAB516 Fab CD40 complex dataset was processed in the space group C2 with the following unit cell dimensions: a = 254.8 b = 224.0 c = 111.4 β = 98.0. A maximum likelihood molecular replacement solution was determined using the program PHASER [20] using the previously solved ABBV-323 complex structure reported above. Coordinates for 5 Fab/CD40 complexes were found in the asymmetric unit based on the molecular replacement solution. Preliminary refinement of the resulting solution was conducted using REFMAC [22, 23] and the program BUSTER [24]. A partial model for CD40 was built manually using the program COOT [25] and examination of 2Fo-Fc and Fo-Fc electron-density maps. CRD3 was not seen in electron density for all 5 CD40 monomers. Additionally, one Fab molecule (monomers K and M) only had the variable regions fit due to disordered density in the constant region. A final round of refinement was conducted using PHENIX [26].The refinement statistics reported R_free_/R_work_ values of 0.255/0.216.

Ramachandran plots and statistics were calculated using MolProbity in the Validation tools from PHENIX [26]. Plots and outliers are included in Additional file 2: Figures: S2a-c.

The ternary complex model of FAB516/CD40L/CD40 presented in Fig. 9a and b was created using the crystal structures of FAB516/CD40 and 3QD6 [4] as guides. The complex was minimized in the program MAESTRO [27] with Prime loop refinement [28] for HCDR2 S52-G56.

## Additional files

Additional file 1: **Figures S1a, b and c:** (a) Overlay of TNFR antiparallel dimer as observed in PDB 1NCF (in blue) and CD40-ABBV-323 antiparallel dimer (in green). (b) and (c) show the antiparallel orientations with N and C termini labeled. (PPTX 328 kb)

Additional file 2: **Figure S2a, b and c:** Ramachadran plots and outliers for all three structures were generated in the program PHENIX (Molprobity) [26]. (PPTX 558 kb)

## Data Availability

Table 2 lists data collection and refinement statistics from all structures listed above. All structural figures were created in the program Pymol (Schrӧdinger) [29]. All structural coordinates have been deposited to the Protein Data Bank (http://www.rcsb.org) with the following PDB codes: 6PE7, 6PE8, 6PE9. Table 3 lists protein quality characterizations for the reagents used in this study.

## Abbreviations

CD40: Cluster of differentiation 40
CD40L: CD40 ligand
CDR: Complementarity-determining region
CRD: Cysteine-rich domain
FAB: Fragment antigen binding domain
HCDR: Heavy chain CDR
ICAM-1: Intercellular adhesion molecule 1
LCDR: Light chain CDR
mAb: Monoclonal antibody
NF-κB: Nuclear factor κappa light chain enhancer
SEAP: Secreted embryonic alkaline phosphatase
Tev: Tobacco Etch Virus nuclear–inclusion-1 endopeptidase
TNF: Tumor necrosis factor
TRAF: TNF receptor associated factor
