# Supplementary figures and images for: CD40/anti-CD40 antibody complexes which illustrate agonist and antagonist structural switches
Source: BMC Mol Cell Biol. 2019 Aug 5;20:29. doi: 10.1186/s12860-019-0213-4 (PMC6683420; doi:10.1186/s12860-019-0213-4)

## Slide 1
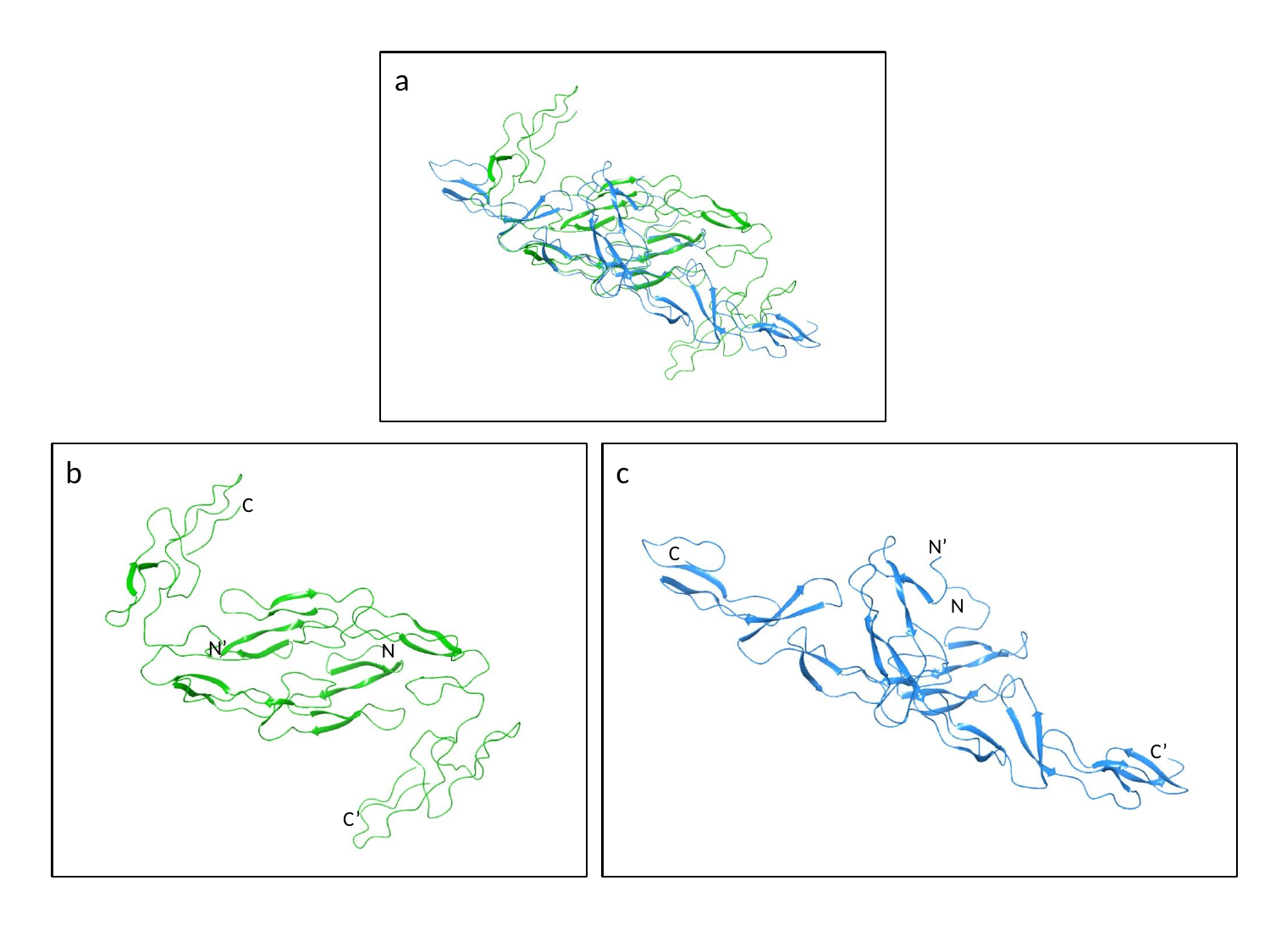

a
b
c
C
N’
C
N
N’
N
C’
C’

Supplement: Supplementary file 1 — Figures S1a, b and c: (a) Overlay of TNFR antiparallel dimer as observed in PDB 1NCF (in blue) and CD40-ABBV-323 antiparallel dimer (in green). (b) and (c) show the antiparallel orientations with N and C termini labeled. (PPTX 328 kb) [file 12860_2019_213_MOESM1_ESM.pptx]
