# Supplementary material for: CD40/anti-CD40 antibody complexes which illustrate agonist and antagonist structural switches
Source: BMC Mol Cell Biol. 2019 Aug 5;20:29. doi: 10.1186/s12860-019-0213-4 (PMC6683420; doi:10.1186/s12860-019-0213-4)
Supplement: Supplementary file 2 — Figure S2a, b and c: Ramachadran plots and outliers for all three structures were generated in the program PHENIX (Molprobity) [26]. (PPTX 558 kb) [file 12860_2019_213_MOESM2_ESM.pptx]

## Slide 1
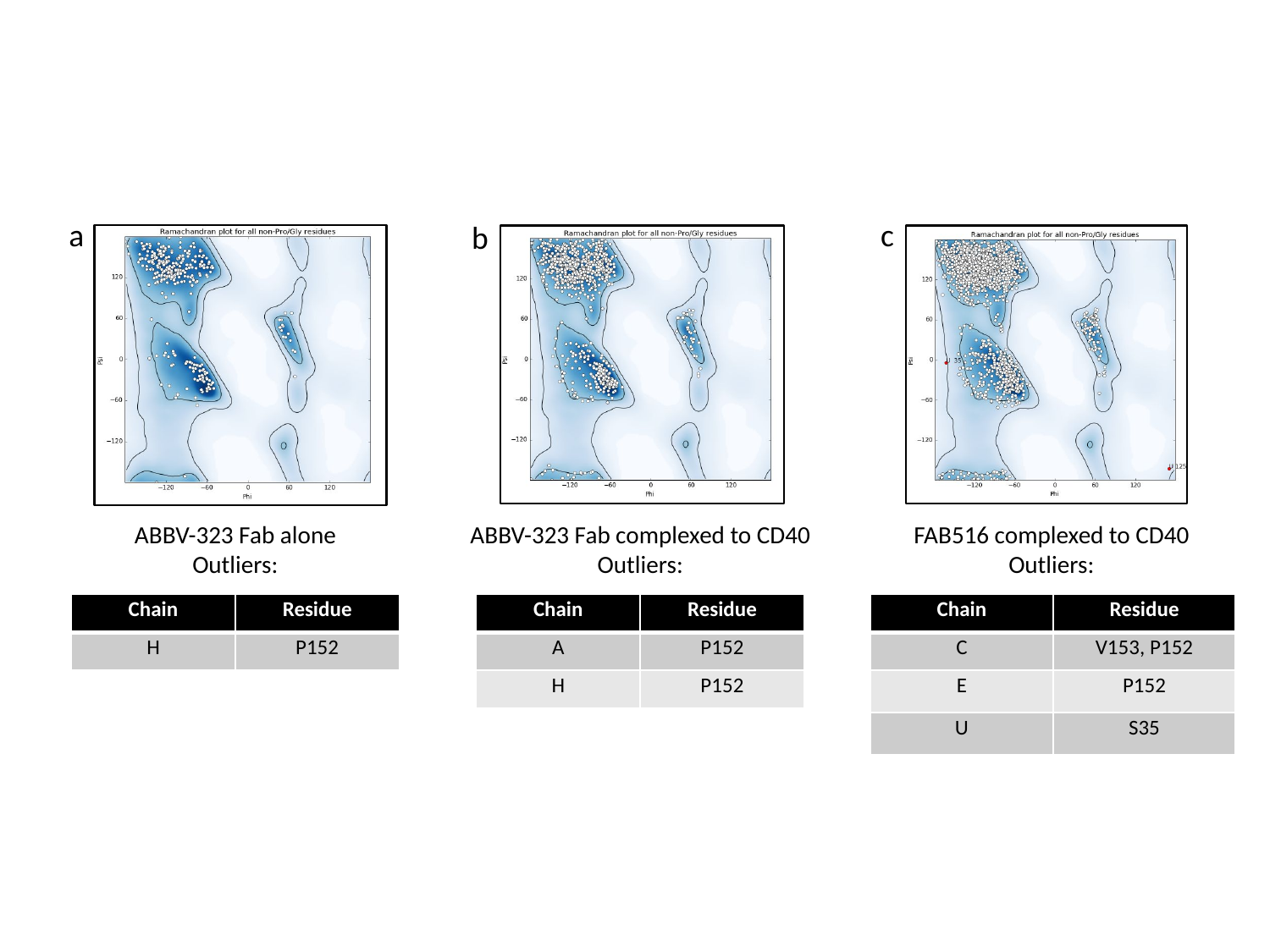

a
c
b
ABBV-323 Fab alone
Outliers:
ABBV-323 Fab complexed to CD40
Outliers:
FAB516 complexed to CD40
Outliers:
| Chain | Residue |
| --- | --- |
| H | P152 |
| Chain | Residue |
| --- | --- |
| A | P152 |
| H | P152 |
| Chain | Residue |
| --- | --- |
| C | V153, P152 |
| E | P152 |
| U | S35 |
